# A distinct parabrachial–to–lateral hypothalamus circuit for motivational suppression of feeding by nociception

**DOI:** 10.1126/sciadv.abe4323

**Published:** 2021-05-07

**Authors:** Siew Cheng Phua, Yu Lin Tan, Alison Maun Yeng Kok, Esra Senol, Christine Jin Hui Chiam, Chun-Yao Lee, Yanmin Peng, Auriel Theodora Jacobea Lim, Hasan Mohammad, Jing-Xuan Lim, Yu Fu

**Affiliations:** 1 Singapore Bioimaging Consortium, Agency for Science, Technology and Research (A*STAR), 138667, Singapore.; 2 Department of Physiology, Yong Loo Lin School of Medicine, National University of Singapore, 117597, Singapore.; 3 Shanghai Key Laboratory of Psychotic Disorders, Shanghai Mental Health Center, School of Medicine, Shanghai Jiao Tong University, Shanghai, China.; 4 Lee Kong Chian School of Medicine, Nanyang Technological University, Singapore.

## Abstract

The motivation to eat is not only shaped by nutrition but also competed by external stimuli including pain. How the mouse hypothalamus, the feeding regulation center, integrates nociceptive inputs to modulate feeding is unclear. Within the key nociception relay center parabrachial nucleus (PBN), we demonstrated that neurons projecting to the lateral hypothalamus (^LH^PBN) are nociceptive yet distinct from danger-encoding central amygdala–projecting (^CeA^PBN) neurons. Activation of ^LH^PBN strongly suppressed feeding by limiting eating frequency and also reduced motivation to work for food reward. Refined approach-avoidance paradigm revealed that suppression of ^LH^PBN, but not ^CeA^PBN, sustained motivation to obtain food. The effect of ^LH^PBN neurons on feeding was reversed by suppressing downstream LH^VGluT2^ neurons. Thus, distinct from a circuit for fear and escape responses, ^LH^PBN neurons channel nociceptive signals to LH^VGluT2^ neurons to suppress motivational drive for feeding. Our study provides a new perspective in understanding feeding regulation by external competing stimuli.

## Teaser

A specific neural circuit for resolving the motivational conflict between feeding and acute pain.

## INTRODUCTION

Feeding is driven by a combination of internal nutritional factors and external factors such as environmental cues or conflicting motivational states (*1*–*5*). Pain, as an evolutionarily conserved signal of threat, poses a substantial competition to feeding behavior, and decreased appetite is one of the most common manifestations of chronic pain (*6*, *7*). While many feeding regulation neuronal populations have been revealed in the hypothalamus, including the orexigenic Agouti-related peptide (AgRP) neurons (*8*), Gamma-Aminobutyric acid (GABA)ergic neurons in lateral hypothalamus (LH) and zona incerta (*9*, *10*), somatostatin neurons in tuberal nucleus (*11*), and other non–AgRP GABAergic neurons in arcuate nucleus (*12*), how and which hypothalamic neurons integrate nociceptive information to modulate feeding behavior have remained elusive. The LH contains many pain-responsive neurons (*13*–*15*), which suggests their potential function in modulating behavioral responses to pain.

During nociception, the parabrachial nucleus (PBN) located in the brainstem is a core en route center transmitting nociceptive signals to higher brain regions that integrate and interpret the sensory information (*16*). PBN projects to diverse brain regions, including the LH (*17*–*20*). Despite robust projection to this hypothalamic region, existing knowledge on the role of PBN-LH communication is limited to a few studies unrelated to pain (*21*, *22*). In the external lateral PBN (elPBN), calcitonin gene–related peptide (CGRP) neurons that project to the central amygdala (CeA) encode for danger signals and regulate threat-related memory and escape responses (*23*–*25*). Tachykinin 1 (Tac1) neurons in the same region have similarly been implicated with encoding escape behavior to noxious heat (*26*). In addition, two populations of PBN neurons have been separately shown to mediate coping responses to sustained pain and itch-induced scratching behavior (*27*, *28*). More recently, spinal nociceptive signals have been demonstrated to be directly channeled to the tachykinin receptor 1 (Tacr1) rather than CGRP neurons in PBN (*29*). Therefore, we hypothesized that the LH neurons could serve as an important hub for integrating nociceptive signals from PBN and modulating feeding behavior.

In this work, we first established that ^LH^PBN (LH-projecting PBN) and ^CeA^PBN (CeA-projecting PBN) neurons are largely nonoverlapping populations and determined the effect of ^LH^PBN neuronal circuit in modulating feeding behavior through reduced motivation to obtain food. We also demonstrated the major downstream target of ^LH^PBN in LH to be VGlut2-positive glutamatergic neurons. Our study thus revealed a distinct neural circuit mechanism for hypothalamic neurons to receive nociceptive signals in modulating feeding behavior, and provided a neural mechanism for pain prioritization.

## RESULTS

### ^LH^PBN neurons are distinct from ^CeA^PBN neurons and respond to acute pain

**Fig. 1:**
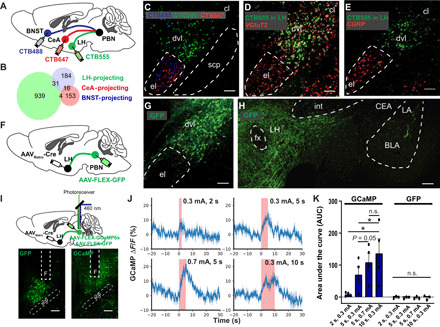
^LH^PBN neurons are segregated from ^CeA^PBN neurons and nociceptive. (**A**) Viral injection of retrograde CTB tracers conjugated with different fluorophores in the indicated brain regions. (**B**) Venn diagram of PBN neurons projecting to LH, CeA, and BNST (number shown in diagram is averaged from eight mice). (**C**) Representative image showing PBN neurons projecting to LH (green), CeA (red), and BNST (cyan) retrogradely labeled as shown in (A). (**D**) After injecting CTB555 retrograde tracer in LH (green), a representative coronal PBN section was stained for *VGluT2* (red) by RNA-FISH. (**E**) After injecting CTB555 retrograde tracer in LH (green), a coronal PBN section was stained for *CGRP* (red) by RNA-FISH. (**F**) Schematic illustrating viral injection strategy enabling GFP expression in ^LH^PBN neurons. (**G**) Representative coronal section showing GFP expressed in ^LH^PBN neurons. (**H**) Representative coronal section showing ^LH^PBN GFP projections in LH but not in CeA. (**I**) Viral injection strategy enabling GFP or GCaMP6s expression in ^LH^PBN neurons. An optic fiber was positioned above the PBN to allow real-time photometric measurements of the fluorescence signal change. (**J**) ^LH^PBN calcium activity in one representative mouse subjected to defined electric shock intensity and duration, revealed by GCaMP6s fluorescence signal change. Graphs were aligned to the start of electric shock. Shock duration is indicated in pink (means ± SEM averaged from triplicate trials). (**K**) Area under the curve (AUC) quantification of fluorescence signal change at defined electric shock intensity and duration. (*n* = 4 and 3 mice for GCaMP and GFP, respectively). BLA, basal lateral amygdala; CeA, central amygdala; cl, central lateral subnucleus; dvl, dorsoventral lateral subnucleus; el, external lateral subnucleus; fx, columns of the fornix; int, internal capsule; LA, lateral amygdala; LH, lateral hypothalamus; PBN, parabrachial nucleus; scp, superior cerebellar peduncle; and *F*, optical fiber. n.s., *P* ≥ 0.05; *P < 0.05. Scale bars, 100 μm. See also table S1 for statistical details.

We first injected retrograde tracer cholera toxin subunit B (CTB) conjugated with different fluorophores into three main PBN projection regions (*25*), namely, the bed nucleus of the stria terminalis (BNST), the CeA, and the LH (Fig. 1A and fig. S1A). Whereas approximately 7 and 13% of ^BNST^PBN (BNST-projecting PBN) neurons coprojected to CeA and LH, respectively, ^LH^PBN and ^CeA^PBN neurons shared a smaller overlap, suggesting distinct neuronal subpopulations (Fig. 1, B and C, and fig. S1B). The spatial enrichment of ^LH^PBN neurons in the dorsoventral lateral PBN (dvlPBN), with partial overlap into the central lateral PBN, was also segregated from ^BNST^PBN and ^CeA^PBN neurons, which mostly located in the elPBN (Fig. 1C and fig. S1B). After retrograde tracing in LH, we detected for *VGluT2* or *CGRP* using RNA fluorescence in situ hybridization (RNA-FISH) and determined ^LH^PBN neurons to be glutamatergic and distinct from CGRP-expressing neurons that project strongly to CeA (Fig. 1, D and E). Further analyses showed that ^LH^PBN neurons could not be fully encompassed by other genetic markers (*Calb1*, *Calb2*, *CCK*, *Pdyn*, and *SST*) expressed in the PBN (fig. S1, C and D) (*25*), rendering it challenging to study ^LH^PBN using single Cre recombinase–expressing transgenic mice. We circumvented this issue by exploiting an adeno-associated virus that retrogradely infects projection neurons through their axon terminals (AAV_Retro_) (*30*). Cre-expressing AAV_Retro_ was injected into the LH to enable Cre expression in ^LH^PBN neurons and further genetic manipulation of these neurons when viruses expressing Cre-dependent genes were injected into PBN (Fig. 1F). Using this strategy, we first expressed green fluorescent protein (GFP) in ^LH^PBN neurons and confirmed that they mainly located in the dvlPBN but projected sparsely to neighboring elPBN (Fig. 1G). Consistent with retrograde tracing results, GFP-expressing ^LH^PBN neurons projected densely to LH but not to CeA (Fig. 1H).

To assess the ability of ^LH^PBN neurons in encoding acute pain information, we expressed genetically encoded calcium indicator GCaMP6s in these neurons and imaged their calcium responses to acute electric shocks using fiber photometry (Fig. 1I). ^LH^PBN neurons showed time-locked responses to electric shocks that were scalable by both the duration and intensity of electric shocks (Fig. 1, J and K). We also examined c-fos expression in the PBN after acute electric shocks and found similar graded responses in total lPBN and ^LH^PBN neurons (fig. S2, A to C). We further demonstrated that ^LH^PBN neurons responded to different modalities of nociceptive signals, including tactile itch and inflammatory pain (fig. S2, D to G), and the cutaneous sensitivity demonstrated by ^LH^PBN neurons was consistent with pseudotyped rabies virus–mediated retrograde tracing revealing monosynaptic connections between these neurons and the dorsal horn of spinal cord (fig. S2, H to K). ^LH^PBN neurons were not robustly responsive to visceral malaise induced by intraperitoneal administration of lithium chloride (LiCl), despite a strong response mounted in elPBN that has been reported to be composed mostly of CGRP neurons (fig. S2, F and G) (*31*). Unlike ^CeA^PBN neurons with an established role in threat memory (*23*, *24*), ^LH^PBN neurons did not respond to auditory cues [conditional stimulus (CS)] associated with 0.7-mA electric shocks [unconditional stimulus (US)] in a CS-US pairing fear-conditioning paradigm (fig. S3, A, B, and E), supporting that they detect pain per se but not pain-associative cues or freezing behavior. We verified that the absence of activity signals was not due to technical reasons, since ^LH^PBN neurons in the same animal responded robustly to acute paw and tail pinches as well as warm water tail dipping at temperatures between 40° and 52°C (fig. S3, C to G). Hence, nociceptive ^LH^PBN neurons constitute a distinct genetic and functional subdivision from ^CeA^PBN neurons.

### Activation of ^LH^PBN neurons suppresses feeding and transmits negative valence

**Fig. 2:**
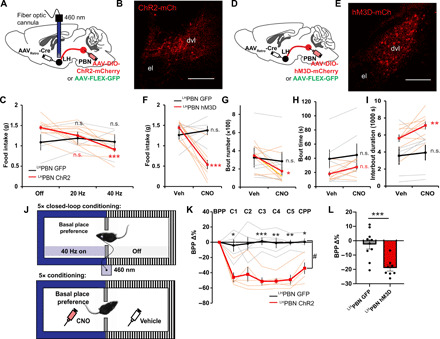
Optogenetic and chemogenetic activation of ^LH^PBN neurons suppress feeding and transmit negative valence. (**A**) Viral injection strategy enabling GFP or Chr2 expression in ^LH^PBN neurons. An optic fiber was positioned in LH to allow optogenetic stimulation of PBN terminals. (**B**) Representative coronal section showing ChR2-mCherry expressed in ^LH^PBN neurons. (**C**) Amount of food consumed in a 3-hour postfasting refeeding experiment with optogenetic stimulation of PBN terminals in LH at indicated frequencies (*n* = 4 and 10 for GFP and ChR2, respectively). (**D**) Viral injection strategy enabling GFP or hM3d-mCherry expression in ^LH^PBN neurons. (**E**) Representative coronal section showing hM3D-mCherry expressed in ^LH^PBN neurons. (**F**) Amount of food consumed in a 3-hour chemogenetic postfasting refeeding experiment with hM3D off (Veh) or on (CNO). GFP served as CNO control [*n* = 6 and 9 for GFP and hM3D respectively; also applies to (G) to (I)]. (**G**) Number of feeding bouts in experiment as in (F). (**H**) The averaged duration of feeding bouts in experiment as in (F). (**I**) The averaged interval duration between feeding bouts in experiment as in (F). (**J**) Top: Schematic illustrating the closed-loop optogenetic conditioned place preference test. Blue light was turned on whenever mice entered the paired zone. Bottom: Schematic illustrating chemogenetic conditioned place preference test. Saline was paired with the less preferred zone; CNO was paired with the basal place preference. (**K**) Percentage change in the basal place preference (BPP) over five conditioning days and test day (*n* = 5 for both GFP and ChR2 groups). (**L**) Percentage change in the BPP after five conditioning days. (*n* = 10 and 8 for GFP and hM3D, respectively). Thick lines or bars represent means ± SEM, and thin lines or dots represent individual mouse. n.s., *P* ≥ 0.05; *P < 0.05; **P < 0.01; and ***P < 0.001. Scale bars, 200 μm. See also table S1 for statistics details.

We proceeded to investigate the effect of ^LH^PBN activation on feeding behavior in overnight-fasted hungry mice. Optogenetic activation of channelrhodopsin-2 (ChR2)–expressing PBN terminals in LH as well as chemogenetic activation of hM3D-expressing ^LH^PBN neurons commonly resulted in reduced total food intake in the fast-refeeding paradigm (Fig. 2, A to F). Feeding pattern analyses revealed that ^LH^PBN specifically reduced the number of eating events by prolonging the interbout duration without interfering mean eating bout time (Fig. 2, G to I), suggesting that ^LH^PBN may limit the motivation to obtain food. This is distinct from the reported effect of ^CeA^PBN activation in limiting eating bout duration (*32*), thereby indicating distinct and complementary roles of ^LH^PBN and ^CeA^PBN in regulating feeding behavior.

We then examined the valence signal transmitted by ^LH^PBN neurons by expressing either GFP or ChR2 in these neurons and investigating the effect of optogenetic stimulation in influencing place preference in a two-zoned chamber test (Fig. 2J). Whereas a 460-nm stimulation of GFP-expressing PBN terminals in LH did not alter place preference after a 5-day closed-loop conditioning paradigm, zone-specific optogenetic stimulation of ChR2-expressing PBN terminals in LH resulted in a reduced time occupancy on null stimulation test day (Fig. 2K), suggesting negative valence transmitted by ^LH^PBN neurons. This conclusion was additionally supported by the chemogenetic place preference test, in which the pairing of ^LH^PBN activation with a basal place preference via CNO injection resulted in a lower occupancy after a 5-day conditioning paradigm (Fig. 2L).

We also examined other aspects of behavioral change upon ^LH^PBN activation to gain a better understanding of its function in modulating behavior. Mice with activated ^LH^PBN neurons spent significantly less time engaged in nesting activity as compared with GFP control mice (fig. S4, A to D). Consistently, movement activity was also reduced in mice with activated ^LH^PBN neurons (fig. S4E). Reduced movement and nesting activity were not due to a more sedentary or freezing state, since activating ^LH^PBN neurons promoted grooming actions (fig. S4F). We further assessed the exploratory behavior of these mice in an elevated plus maze (EPM) (fig. S4G). Compared with GFP control mice, ^LH^PBN-activated mice traveled less and made fewer arm entries (fig. S4, H and I). These mice also showed a tendency to avoid the EPM open arms, and the time spent in open arm in each entry was markedly reduced (fig. S4, J and K). In contrast, chemogenetic attenuation of ^LH^PBN neurons led to a converse increase in the time spent in the EPM open arms, indicating bidirectional control of exploratory behavior by these neurons (fig. S4, L to O). Together, these results showed that reduced food consumption after activating ^LH^PBN neurons was not due to a sedentary or freezing state, but rather a potential loss of motivation to obtain food.

### Activation of ^LH^PBN neurons represses motivation to work for food rewards

**Fig. 3:**
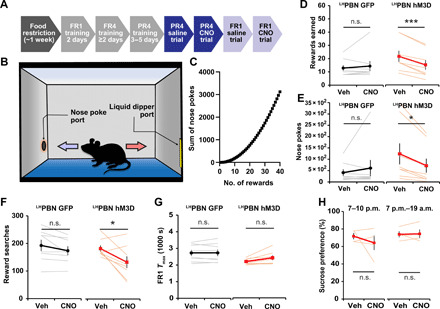
^LH^PBN neuron activation represses performance in food reward operant task. (**A**) Schematic illustrating the timeline for operant task training and testing. (**B**) Schematic illustrating the setup for operant task; the nose poke port and liquid dipper port were located at diagonal corners of the experiment chamber. (**C**) Graph shows the sum of nose pokes required for gaining successive rewards on a progressive ratio 4 (PR4) task. (**D**) The number of rewards earned in a 1-hour PR4 task with hM3D off (Veh) or on (CNO). GFP served as CNO control. [*n* = 8 for both GFP and hM3D groups; also applies to (E) to (G)]. (**E**) Number of nose pokes accumulated in the experiment as in (D). (**F**) Number of searches for reward in the experiment as in (D). A reward search is defined as a single trip from nose poke port to liquid dipper port. (**G**) Time needed to gain maximum number of rewards (60) on FR1 task, with Veh or CNO for respective groups. (**H**) Quantification of sucrose preference in indicated time periods with Veh or CNO for respective groups (*n* = 4 for both 3- and 14-hour trials). Thick lines represent means ± SEM, and thin lines represent individual mouse. n.s., *P* ≥ 0.05; *P < 0.05; and ***P < 0.001. See also table S1 for statistical details.

To directly test whether the suppressed food intake upon ^LH^PBN activation was due to a reduced motivational drive, we trained mice to perform an operant task in which they learned to perform nose pokes in return for food rewards (Fig. 3, A and B). In a progressive ratio (PR) operant test, where an increasing number of nose pokes were required to earn each subsequent reward (Fig. 3C), ^LH^PBN activation reduced the number of rewards earned, while clozapine N-oxide (CNO) injection in GFP control mice did not elicit a significant change (Fig. 3D). Consistently, ^LH^PBN-activated mice performed fewer numbers of nose pokes and reward searches when comparing vehicle and CNO trials (Fig. 3, E and F); these results suggested that the activation of ^LH^PBN represses the motivation of mice to work for rewards. To verify that the reduced performance induced by ^LH^PBN activation was not owing to a lower perceived value of food, we further tested mice on a low-effort fixed ratio 1 (FR1) schedule, in which each reward requires a single nose poke (Fig. 3A). In this task, the two groups of mice spent comparable amounts of time in obtaining the maximum reward regardless of vehicle or CNO administration, and the ability of ^LH^PBN-activated mice to move at matched rates with the control mouse group substantiates that the former did not suffer from locomotion defects (Fig. 3G). Consistently, ^LH^PBN activation also did not lead to a change in sucrose preference (Fig. 3H). Hence, the activation of ^LH^PBN neurons did not change the perceived value of reward but rather repressed motivation to work for reward.

### Quantitative analysis of approach-avoidance conflict involving acute shock pain

With independent demonstration of ^LH^PBN acute pain responses and the effect of these neurons on feeding motivation, we hypothesized that acute nociception may acquire ^LH^PBN activity to suppress feeding. Inspired by the pioneering work of Miller and others (*33*, *34*) in approach-avoidance conflicts, we established an experimental paradigm that could elicit characteristic approach-avoidance conflict behaviors in mice.

Since hunger satiation by feeding is rewarding (*35*), we initially presented hunger as a competing state with pain induced by electric shocks. Overnight-fasted hungry mice were placed in a dual-region chamber; liquid food was located in a region floored with metal grid delivering continuous electric shock, whereas the adjacent region had no food but was shielded from shocks via insulative flooring (fig. S5A). In 0-mA shock-off trials, hungry mice quickly accessed the metal grid and consumed food within a 15-min time period, while application of a low electric intensity of 0.12 mA during shock-on trials was sufficient to limit hungry mice from entering the metal grid and accessing food (fig. S5, B and C). Although both mild (0.12 mA) and stronger electric shocks (0.7 mA) elicited similar effect on food consumption amount (fig. S5, B and C), mice demonstrated very different behavioral dynamics revealed by their position heatmaps in the chamber (fig. S5, D and E). Closer examination revealed characteristic vacillation behavior at the border between the metal grid and insulated regions that was manifested as the decision-action fluctuation between reward approach (advance into grid region) and pain avoidance (retreat into insulative region) (fig. S5A and movie S1); this vacillation behavior could be quantitatively analyzed by tracking and analyzing the advance/retreat movements of mice (fig. S5G). Whereas mild electric shock (0.12 mA) resulted in noticeable vacillation behavior within the decision zone, as revealed by repeated retreating events in decision zone, strong electric shock (0.7 mA) that likely resulted in intense fear greatly repressed vacillation behavior and was associated with more time spent in the rest zone (fig. S5, E to H). Our behavioral analysis also showed the generalization of approach-avoidance behavior in different reward conflict scenarios (fig. S5, I to N, and movies S2 to S4). These results demonstrated that we could quantitatively analyze approach-avoidance behavior using this paradigm and we were prompted to use a 0.12-mA shock as an aversive cue in subsequent experiments, since it elicited vacillation behavior characteristic of an approach-avoidance motivational conflict.

### Attenuating ^LH^PBN promotes effort to obtain food in approach-avoidance conflict

**Fig. 4:**
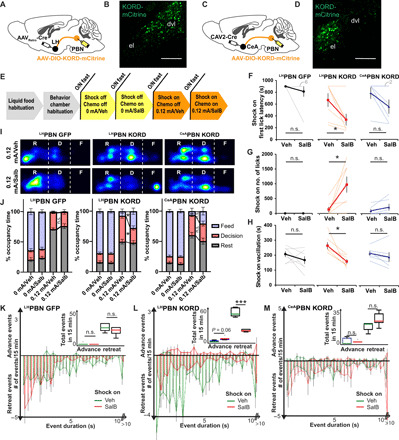
Suppressing ^LH^PBN but not ^CeA^PBN drives food consumption in the presence of pain caused by mild electric shocks. (**A**) Viral injection strategy to enable the KORD-mCitrine expression in ^LH^PBN neurons. (**B**) Representative coronal sections showing the KORD-mCitrine expression in ^LH^PBN neurons. (**C**) Viral injection strategy to enable the KORD-mCitrine expression in ^CeA^PBN neurons. (**D**) Representative coronal sections showing the KORD-mCitrine expression in ^CeA^PBN neurons. (**E**) The order of trials in pain-reward (liquid food) conflict paradigm, shock-off (0 mA) and shock-on (0.12 mA). SalB was injected 30 min before each experiment. (**F**) Time latency to first lick at lickometer during shock-on trials. Latency was set as 900 s for mice that did not lick during the 15-min trial [*n* = 7, 7, and 6 mice for GFP, ^LH^PBN KORD, and ^CeA^PBN KORD, respectively; also applies to (G), (H), and (J)]. (**G**) Number of licks at the lickometer port accumulated within 15 min during shock-on trials. (**H**) The amount of time spent vacillating in the decision zone during shock-on trials. (**I**) Heatmap visualization of location frequencies across “rest (R),” “decision (D),” and “feed (F)” zones of one representative mouse at indicated trials. (**J**) Average time occupancy in rest, decision, and feed zones at indicated trials, expressed as a percentage. (**K** to **M**) Histogram depicts the distribution of advance and retreat events at the decision zone according to event duration by mice expressing (K) GFP or (L) KORD in ^LH^PBN or (M) KORD in ^CeA^PBN in respective shock-on trials. Inset shows the box plot of advance and retreat events in 15-min trials [*n* = 5 for both Veh and SalB trials for (K) and (L); *n* = 6 for both Veh and SalB trials for (M)]. Thick lines or bars represent means ± SEM, and thin lines or dots represent individual mouse. n.s., *P* ≥ 0.05; *P < 0.05; and ***P < 0.001. Scale bars, 200 μm. See also table S1 for statistical details.

To dissect the roles of ^LH^PBN and ^CeA^PBN in suppressing food consumption by mild acute pain, we expressed G_i_-coupled designer receptors exclusively activated by designer drugs (DREADD), κ-opioid DREADD (KORD), in these neurons to enable inducible neuronal attenuation by salvinorin B (SalB) (Fig. 4, A to D) (*36*). We then investigated how functional suppression of these neurons affected behavioral decisions in hungry mice subjected to shock-off and shock-on trials in our approach-avoidance conflict paradigm (Fig. 4E). Whereas chronic silencing of ^CeA^PBN neurons was reported to promote food intake in the time scale of hours (*32*), acute ^CeA^PBN neuronal attenuation induced by SalB during shock-off trials did not significantly affect food intake during a 15-min time period (fig. S6E, blue lines). In contrast, mice expressing KORD in ^LH^PBN consumed more food in shock-off trials upon SalB administration (fig. S6E, red lines) that was associated with a reduced number of retreating events in the decision zone (fig. S6H).

During shock-on vehicle trials, the introduction of continuous 0.12-mA electric shock significantly shifted occupancy toward the rest and decision zones in all three groups of mice, denoting the strong feeding suppression in these mice (Fig. 4, I and J, and fig. S6, A to C). SalB administration in mice expressing KORD in ^LH^PBN reduced the latency of food intake and promoted food intake in shock-on trials, while no significant change was observed for the other two groups of mice (Fig. 4, F and G). To better understand this behavioral change, we analyzed the vacillation kinetics in these mice. In mice expressing KORD in ^CeA^PBN, SalB induced a shift in time occupancy in the rest zone toward the feeding zone while maintaining the time spent in the decision zone (Fig. 4J). This was likely due to a delayed tendency to escape whenever mice landed on the shock grid in the feeding zone (movie S5) and is consistent with the reported role of ^CeA^PBN neurons in driving escape behavior (*24*). While this extended the amount of time spent in the feeding zone (Fig. 4J) and reduced latency to food access in some mice (Fig. 4F, blue lines), pain that resulted from mild electric shock likely still impeded motivation of food consumption such that overall food intake was unchanged (Fig. 4G, blue lines). In contrast, the higher feeding zone occupancy in ^LH^PBN KORD-expressing mice during the SalB trials was associated with a significant drop in the time spent in the decision zone (Fig. 4, I and J). Together with a shorter vacillation time (Fig. 4H, red lines) and a markedly reduced number of retreating events (both not observed in control or ^CeA^PBN groups) (Fig. 4, K to M), these observations support that acute attenuation of ^LH^PBN neurons resolved the motivational conflict between food consumption and mild acute pain. In contrast, attenuating ^LH^PBN neurons did not rescue appetite suppression caused by LiCl-induced visceral malaise (fig. S7B), an effect that was reported to be partially rescued by inhibiting CeA-projecting CGRP neurons in PBN (*37*). This result is consistent with our finding that LiCl strongly activated neurons located in elPBN but not ^LH^PBN neurons (figs. S2F and S7C) (*37*).

To verify whether the appetitive effect of attenuating ^LH^PBN neurons in the presence of mild acute pain was due to a dampened physical sensation, we performed the von Frey test and confirmed that neither attenuating nor activating ^LH^PBN neurons altered paw tactile sensitivity (fig. S8A). Furthermore, we showed that attenuating ^CeA^PBN rather than ^LH^PBN significantly repressed the escape motion to 0.3- and 0.7-mA shocks (fig. S8B), consistent with the reported role of ^CeA^PBN in mediating threat-associated escape behavior (*24*). When an alligator clip was applied to the hind paw to elicit short periods (60 s) of sustained pain (fig. S8C) (*27*), ^LH^PBN-suppressed mice spent significantly less time licking their affected paw as compared with GFP control mice and ^CeA^PBN-attenuated mice, supporting a role for ^LH^PBN circuit in pain attendance (fig. S8D) (*27*). Through quantitative and extensive behavioral analyses, our observations corroboratively demonstrated that ^LH^PBN neurons, but not ^CeA^PBN neurons, are responsible for suppressing the effort to obtain food in a motivational conflict caused by mild acute pain, further strengthening the role of ^LH^PBN neurons in modulating feeding motivation.

### ^LH^PBN neurons form monosynaptic glutamatergic synapses with LH^VGlut2^ neurons

**Fig. 5:**
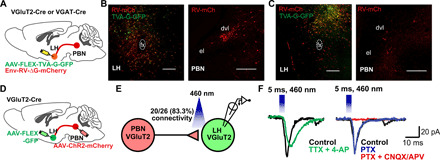
^LH^PBN neurons send monosynaptic glutamatergic input to LH^VGlut2^ neurons. (**A**) Schematic illustrating the pseudotyped rabies virus injection strategy that retrogradely labels input neurons to glutamatergic (VGluT2) or GABAergic (VGAT) neurons in LH using VGluT2-Cre mice (B) or VGAT-Cre mice (C). (**B**) Left: Representative LH coronal section showing starter cells (yellow) for the RV-mCh retrograde labeling of LH^VGluT2^ neuron inputs. Right: Representative PBN coronal section showing the RV-mCh–labeled neurons in PBN that projected to LH^VGluT2^ neurons. (**C**) Left: Representative LH coronal section showing starter cells (yellow) for RV-mCh retrograde labeling of LH^VGat^ neuron inputs. Right: Representative PBN coronal section showing the RV-mCh–labeled neurons in PBN that projected to LH^VGat^ neurons. (**D**) Schematic illustrating the virus injection strategy to express ChR2-mCh in PBN neurons and GFP in LH^VGluT2^ neurons for electrophysiology examination of the synaptic connection between PBN and LH glutamatergic neurons. (**E**) Diagram of electrophysiology experiment in acute hypothalamic slices for examining the connection between PBN and LH glutamatergic neurons. In 26 recordings performed on four animals, 20 glutamatergic neurons in LH showed time-locked response to blue light illumination. (**F**) Representative electrophysiology traces confirming that the connection between PBN and LH glutamatergic neurons is monosynaptic and glutamatergic. (TTX, tetrodotoxin; 4-AP, 4-aminopyridine; PTX, picrotoxin; CNQX, 6-cyano-7-nitroquinoxaline-2,3-dione; and APV, d-2-amino-5-phosphonopentanoic acid).

While we have revealed the function of PBN neurons that project to the LH, the major downstream targets of ^LH^PBN neurons remain unclear. Accordingly, we examined the monosynaptic inputs of major LH neuronal subtypes using pseudotyped rabies virus–mediated retrograde tracing. In VGlut2-Cre mice, the injection of Cre-dependent TVA and G proteins in LH^VGlut2^ neurons (Fig. 5A) confirmed that LH^VGlut2^ glutamatergic neurons received monosynaptic inputs from dvlPBN (Fig. 5B). Yet, targeting GABAergic or orexinergic neurons in LH (using VGAT-Cre or orexin-Cre mice, respectively) via similar viral tracing strategies revealed a much weaker connectivity between dvlPBN and LH GABAergic [expressing vesicular GABA transporter (VGAT)] or orexin hypocretin (HCRT) neurons (Fig. 5C and fig. S9, A and B). Furthermore, whole-cell recording in acute hypothalamic slices expressing GFP in LH^VGlut2^ neurons and ChR2-mcherry in dvlPBN VGlut2-positive neuron terminals (Fig. 5D) confirmed that approximately 83% of the recorded glutamatergic VGlut2-positive neurons in LH received monosynaptic glutamatergic inputs from PBN^VGluT2^ neurons (Fig. 5, E and F). RNA-FISH analysis further revealed that optogenetic stimulation of ChR2-expressing PBN terminals in the LH mostly activated *VGluT2*-expressing glutamatergic neurons in this hypothalamic region (fig. S9, C to E).

### ^LH^PBN neurons suppress feeding through activating downstream LH^VGlut2^ neurons

**Fig. 6:**
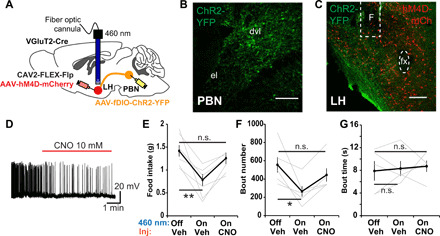
The effect of LH^VGlut2^ neurons on feeding depends on downstream ^LH^PBN neurons. (**A**) Schematic illustrating the virus injection strategy to enable ChR2 expression in ^LH^PBN glutamatergic neurons and hM4D in LH glutamatergic neurons. A fiber optic cannula was positioned in LH to allow blue light optogenetic stimulation of PBN terminals. (**B**) Representative coronal section showing the ChR2-YFP expression in ^LH^PBN based on the virus injection strategy in (A). (**C**) Representative coronal section showing the ChR2-YFP–expressing PBN terminals in LH and hM4D-mCh expression in LH^VGluT2^ neurons based on the virus injection strategy in (A). F denotes fiber optic cannula position. (**D**) Representative trace of a whole cell–patched LH glutamatergic neuron expressing hM4D-mCherry with CNO application (red line) into bath of recording chamber. (**E**) Amount of food consumed in 3-hour feeding experiment postovernight fasting. Each mouse was subjected with three consecutive experiment trials on different days: (i) 460 nm–off hM4D-off (Veh), (ii) 460 nm–on hM4D-off (Veh), (iii) 460 nm–on hM4D-on (CNO) (*n* = 6). (**F**) Number of feeding bouts in experiment as in (E) (*n* = 5). (**G**) The mean time duration of all feeding bouts in experiment as in (E) (*n* = 5). Thick lines represent means ± SEM, and thin lines represent individual mouse. n.s., *P* ≥ 0.05; *P < 0.05; **P < 0.01. Scale bars, 200 μm. See also table S1 for statistical details.

We lastly examined whether the feeding effects of ^LH^PBN neurons depends on downstream LH^VGlut2^ neurons. Flp recombinase–dependent ChR2 was expressed in LH-projecting glutamatergic PBN neurons based on the injection of retrogradely transporting canine adenovirus type 2 (CAV2)-FLEX-Flp virus in the LH of VGlut2-Cre mice, and inhibitory DREADD hM4D was coexpressed in LH of VGlut2-Cre mice; this allowed for simultaneous optogenetic stimulation of ^LH^PBN neuron terminals and chemogenetic inhibition of LH^VGlut2^ neurons (Fig. 6, A to D). As expected, stimulation of ChR2-expressing terminals of ^LH^PBN neurons strongly suppressed feeding after overnight fasting by reducing eating frequency but not mean eating bout duration [Fig. 6, E to G, vehicle (Veh) injection groups]. Concurrent CNO injection in these mice to suppress LH^VGlut2^ neurons significantly reversed the feeding effect of optogenetic stimulation, precisely by enhancing eating frequency without a change in mean eating bout duration (Fig. 6, E to G, CNO groups). As a control, chemogenetic suppression of LH^VGlut2^ neurons had no significant effect on feeding in food-restricted mice (fig. S10). Thus, we demonstrated a necessary role of LH glutamatergic neurons in ^LH^PBN-mediated feeding suppression.

## DISCUSSION

While significant efforts have been invested in understanding how hypothalamic neurons sense nutritional signals to modulate feeding behavior (i.e., AgRP and tuberal nucleus somatostatin neurons that sense ghrelin in hunger state) (*3*, *11*), feeding also engages a dynamic decision-making process strongly influenced by many external factors, including pain, that extend beyond fulfilling nutritional demands (*1*–*5*). While danger-encoding CGRP neurons in PBN have been found to suppress feeding through their projection to the CeA (*24*, *25*, *37*), it was unclear how hypothalamic neurons integrate nociceptive signals to modulate feeding behavior, likely through non–CGRP PBN neurons that receive direct nociceptive inputs (*29*). Combining in vivo fiber photometry imaging and c-fos staining, we demonstrated that ^LH^PBN neurons respond to nociceptive inputs (although a potential response to arousal was not completely ruled out). Our study has revealed an unexpected role of ^LH^PBN neurons in transducing nociceptive signals to LH^VGlut2^ neurons and robustly suppressing feeding behavior in a manner complementary to CGRP neurons (regulating eating frequency versus bout duration).

We have performed extensive behavioral experiments to demonstrate that ^LH^PBN activation reduces motivation in mice to obtain food. In agreement with an earlier report that demonstrated lateral habenula (LHb)–projecting glutamatergic LH neurons in limiting food consumption (*38*), we confirmed PBN-LH^VGluT2^-LHb connectivity using pseudotyped rabies virus–tracing strategy (fig. S9, F and G). Since the LHb is a core brain region implicated in depression that mediates avoidance behavior (*39*–*41*), it is likely that it also mediates the withdrawal of motivation to obtain reward (*42*) as observed in ^LH^PBN-activated mice. Despite no apparent relation with feeding, we also found that activating ^LH^PBN neurons increased grooming behavior, which likely involved LH^VGluT2^ neurons projecting to the paraventricular hypothalamus (*43*).

Pioneered by Miller and others (*33*, *34*), the approach-avoidance paradigm has been extensively used for studying motivational conflicts (*44*). In this study, we further developed this behavioral paradigm and provided a more detailed description of conflicting motivational states by quantifying dynamic retreating or vacillation behavior (fig. S5). Using such quantitative behavioral analysis, we discovered that pain resulting from mild electric shock (0.12 mA) elicited a very different behavioral outcome from stronger shock (0.7 mA or higher), which likely elicited additional fear and escape responses, rendering food or other rewards with insufficient drive for competition. Results from this analysis also suggest that simply measuring food intake under different pain intensities may not be sufficient in understanding the behavioral mechanism underlying appetite change. While food consumption was almost completely suppressed under mild electric shock (0.12 mA), hungry mice demonstrated extensive vacillation behavior that indicated failed attempts to overcome pain and obtain food (fig. S5). To our surprise, only suppressing ^LH^PBN but not ^CeA^PBN enhanced food intake in this conflicting scenario, likely reflecting the more prominent role of ^LH^PBN in motivational drive for feeding. In contrast, CGRP ^CeA^PBN neurons have a more significant role in controlling escape responses to stronger electric shocks (fig. S8B) (*24*). In addition to a recent study reporting non–CGRP Tacr1 neurons in PBN as a major direct target of spinal nociceptive signals (*29*), we showed that ^LH^PBN neurons similarly receive direct neural inputs from the spinal cord (fig. S2, H to K). Together, these findings suggest that mild acute pain that does not elicit fear (*45*, *46*) can be efficiently transmitted from the spinal cord to the LH via the non–CGRP ^LH^PBN neurons and modulate motivational drive for feeding. The ^LH^PBN neurons also overlap with Pdyn neurons in PBN (which also project extensively to LH) that have been recently shown to function as a key hub in sensing mechanosensory inputs and controlling ingestion behavior (*47*). Therefore, ^LH^PBN and ^CeA^PBN neurons constitute two distinct circuits that modulate different aspects of feeding behavior in response to nociceptive signals (feeding motivation versus satiety/fear). Further studies revealing input modulation to ^LH^PBN and ^CeA^PBN neurons will advance our knowledge of how different aspects of pain are regulated. Neural circuit mechanisms underlying different types of motivational conflicts are still not fully understood, and our results provide a new perspective in understanding how pain is prioritized over other conflicting states (i.e., hunger).

Last, our work underscores the complexity of neural pathways mediating the complete nociceptive experience. Hypervigilance to pain has been proposed as a maladaptation in chronic pain that results in a heightened pain perception as well as an unending need to eliminate pain (*48*). While clinical psychologists have proposed that chronic periods of pain elicit emotional fear to drive hyperattention to pain-related information (*49*), recent motivational accounts of pain have highlighted a tendency for chronic pain sufferers to set pain control as an adaptive goal that would, in turn, drive the detection and processing of pain-related information (*48*). It is tempting to speculate a role of the ^LH^PBN pathway in enabling such motivational reinforcement of pain hyperattention in chronic pain sufferers. Furthermore, the ability of ^LH^PBN to regulate motivational drive in obtaining food reward may also be pertinent to the development of comorbid depressive disorders that often accompany chronic pain (*50*). Accordingly, reduced motivation was found in rodent models of neuropathic chronic pain (*51*), and these animals showed hyperactivity in the lateral PBN (*52*). Moreover, while analgesic drugs are critical in reducing uncomfortable feelings associated with pain, they were not effective in reversing reduced motivation in chronic pain animal models (*51*). Likewise, commonly used analgesic buprenorphine (BP) failed to rescue feeding suppressed by either acute foot shocks (fig. S11A) or ^LH^PBN activation (fig. S11D) and did not prevent ^LH^PBN neurons from being activated by foot shock pain (fig. S11, E and F). Instead, BP markedly reduced appetite (fig. S11D, mCherry group) and induced a behavioral state characterized by hyperactivity (fig. S11, G and H) (*53*). Together, these observations warrant the importance of pursuing a role for LH-projecting parabrachial neurons and developing better analgesics in fighting against the psychological maladaptive deployment of chronic pain diseases.

## MATERIALS AND METHODS

### Experimental animals

All experiment procedures were approved by the Institutional Animal Care and Use Committee of the Agency for Science, Technology and Research (A*STAR), Singapore. Animals were housed in a standard 12-hour light-dark cycle animal facility with ad libitum access to water and chow. Male mice between 2 and 8 months of age were used in most experiments. Wild-type C57BL/6J mice were purchased from InVivos Pte Ltd. (mice originally sourced from the Jackson laboratories), VGLUT2-IRES-Cre (stock no. 016963) and VGAT-IRES-Cre (stock no. 016962) mice were purchased from the Jackson laboratories, and HCRT-IRES-Cre mice were generated for our lab by Cyagen Biosciences.

### Stereotaxic surgeries

Standard stereotaxic procedures were carried out on mice anesthetized with ketamine/xylazine cocktail (ketamine: 75 mg/kg, xylazine: 10 mg/kg). BP analgesic was provided during surgery, and Antipam was used as postsurgery reversal drug. Dexamethasone was additionally given in fiber optic cannula implant procedures to reduce inflammation. During surgery, the mouse skull was exposed via a small scalp incision, and craniotomy was performed using a 0.6-mm drill tip. Viruses or 0.25% Alexa fluorophore–tagged CTB retrograde tracers (Invitrogen) were delivered at 2 nl/s with a Micro4 syringe pump (World Precision Instruments) and 10-μl syringe fitted with 33-gauge needles (NanoFil). Six-millimeter fiber optic cannulae [200-μm core, numerical aperture (NA) 0.37, Newdoon] were used for optostimulation in LH, while 4-mm fibers were used for photometric measurements in PBN. Retro-Cre viruses [mixed with CTB (0.25 mg/ml) in a 5:1 ratio] were injected 2 weeks before viral injections delivering Cre-dependent genes, while canine adenovirus type 2 (CAV2)-Cre viruses [mixed with CTB (0.25 mg/ml) in a 5:1 ratio] may be injected with Cre-dependent viruses on the same day. EnvA G–deleted Rabies-mCherry (EnvA-RV-ΔG-mCherry) viruses were injected 2 weeks after AAV8-EF1a-FLEX-GTB (FLEX-TVA-G-GFP) virus injections, and mice were euthanized 7 to 10 days afterward for retrograde tracing in specific brain regions. Similarly, mice used in the CTB retrograde–tracing experiments were euthanized 1 to 2 weeks after CTB injections. Otherwise, mice were allowed to recover for three or more weeks in the animal facility before behavior experiments. Stereotaxic coordinates used for injections or implantations in specific brain regions are listed below (AP, anterior-posterior; ML, medial-lateral; and DV, dorsal-ventral):

LH: AP, between −1.1 and −1.3 (depending on bregma-lambda distance); ML, ±1.15; and DV, −4.9 (for injections) or −4.7 (for optic fiber implants)

PBN: AP, between −5.0 and −5.5 (depending on bregma-lambda distance); ML, ±1.3 to 1.35; DV, −2.5 (for injections) or −2.4 (for optic fiber implants)

CeA: AP, between −0.85 and −0.90 (depending on bregma-lambda distance); ML, ±2.4; and DV, −4.45.

The following lists viruses used in this study: rAAV-retro-CAG-Cre recombinase [University of North Carolina (UNC)], CAV2-Cre recombinase (Plateforme de Vectorologie de Montpellier), CAV2-FLEX-Flp (Plateforme de Vectorologie de Montpellier), AAV1-pCAG-FLEX-EGFP-WPRE (Addgene), AAV8-hSyn-DIO-hM3D(G_q_)-mCherry (Addgene), AAV8-hSyn-DIO-hM4D(G_i_)-mCherry (Addgene), AAV8-hSyn-dF-HA-KORD-IRES-mCitrine (Addgene), AAV5-EF1a-DIO-hChR2(H134R)-mCherry (UNC), AAV5-CaMKIIa-hChR2(H134R)-mCherry (Addgene), AAVdj-EF1-fDIO-hChR2-YFP-WRPE (UNC), AAV9-Syn-FLEX-GCaMP6s-WPRE-SV40 (Addgene), AAV8-EF1a-FLEX-GTB (Salk), EnvA G–deleted Rabies-mCherry (Salk), AAV8-CAG-FLEX(FRT)-TVA-mCh (Salk), AAVdj-CAG-fDIO-oG (Salk), and EnvA G–deleted Rabies-GFP (Salk).

### Acute pain–reward motivational conflict experiments

These experiments were conducted in a sound-attenuated fear-conditioning chamber (Med Associates Inc.) with two-third area of the metal grid flooring (grid region) covered by an acrylic board (insulated region). In liquid food experiments, a measurement bottle containing liquid food (Ensure, Abbott Laboratories) was installed in a wall behind the grid region so that mice have to enter the grid region to access food. Mouse diet in the home cage was converted from regular chow to liquid food 2 days earlier to habituate mice to the novel liquid diet. On the third day, mice were habituated for 1 hour in the experiment chamber with ad libitum liquid food access to familiarize with food location. On the fourth day, liquid food was removed from the home cage at 10:00 a.m., and mice were granted a 15-min session between 10:00 a.m. and 1:00 p.m. the following day with free liquid food access in the chamber to accustom them to fast-refeed operation. The 10:00 a.m.–fast 10:00 a.m.–refeed regime was used in all experiments. Separate batches of mice were used for the 0-, 0.12-, and 0.7-mA trials. Each trial lasted 15 min, and specified electric shocks were delivered continuously from the metal grid flooring. For experiments in Fig. 4, each mouse was subjected to four trials carried out in the following order, with at least 1 day of rest in between: (i) shock-off (0 mA) chemo-off (Veh), (ii) shock-off (0 mA) chemo-on (SalB), (iii) shock-on (0.12 mA) chemo-off (Veh), and (iv) shock-on (0.12 mA) chemo-on (SalB). Vehicle or SalB [10 mg/kg in dimethyl sulfoxide (DMSO); Sigma-Aldrich] was subcutaneously injected in mice 30 min before experiment. Each trial lasted 15 min, and specified electric shocks were delivered continuously from the metal grid flooring. The number and timing of food licks were recorded by an infrared photobeam lickometer and Med-PC software (Med Associates Inc.). For experiments assessing the analgesic effect of BP, BP (0.1 mg/kg) or saline was intraperitoneally administered an hour before each trial.

For experiments involving chow diet, water, and novel female access, the insulative region consisted of an acrylic board with smaller area, and cues were located in the grid region at approximately 10 cm away from the insulative region. In these experiments, separate batches of mice were subjected to shock-off (0 mA) or shock-on (0.12 mA) trials. In both chow and water experiments, the 10:00 a.m.–fast 10:00 a.m.–refeed regime was applied. In the novel female experiment, a 2-month-old virgin female mouse was enclosed in a plastic grid box with a base that shields from electric shocks, and a different mouse was used in each trial. Each trial lasted 15 min, and specified electric shocks were delivered continuously from the metal grid flooring. The amount of food consumed was scored by comparing the weight of the chow before and after the experiment. The latency to reward access and the time spent licking water or social interacting (poking nose into the cage housing novel female) were manually scored by viewing videos recorded from trials.

All experiments were recorded with near-infrared (NIR) video camera with NIR in-house lighting. As illustrated in fig. S4A, vacillation time was obtained by manually observing and scoring the duration each mouse spent moving back and forth along the shock-insulative region border in the decision zone. Timer was started when mouse approached the region border. Care was taken to only consider the time when the head of the mouse is facing the reward zone, and we further excluded the time when mouse was solely engaged in grooming activity at the border. The timer was stopped when mouse returned to the rest zone or when the mouse advanced its body completely into the metal grid region. Zone occupancy times and location frequency heatmaps were automatically generated by motion tracking function in EthoVision XT 12. A Python-based motion tracking program (Tracktor; https://github.com/vivekhsridhar/tracktor) (*54*) was customized to automatically track and score the vacillation behavior at the decision zone. Specifically, for modifying the Tracktor main program, we cropped the video so that only the part in which mouse moves is analyzed (frame = new_frame[165:235,1:255]); we set a threshold so that any value above it is set to be bright because the mouse is dark (frame[np.where(frame>58)] = 255) and blurred the image using a Gaussian filter [frame = ndimage.gaussian_filter(frame,7.6)] so that thin grids with dark shadow are filtered out.

### Operant learning task

The operant learning task was conducted as previously described (*51*) in a modified fear-conditioning chamber with white acrylic board as the flooring. Nose poke port and liquid dipper port delivering liquid food rewards (Ensure, Abbott Laboratories) were installed in diagonal corners of the chamber. Light-emitting diode (LED) light in the nose poke port cues the mice for nose pokes but turns off during time-out periods. Before operant task training, mice were single housed and food restricted to attain 85 to 90% of the free feeding weight. Food-restricted mice were first trained on an FR1 schedule for 2 days, in which each nose poke triggers the delivery of 0.01 ml of liquid food reward with a time-out period of 30 s. This was followed by at least 2 days of the FR4 schedule (four nose pokes triggers the delivery of one reward with a time-out period of 20 s) until mice obtain a maximum of 60 rewards. Next, mice were trained on a PR4 schedule in which the nose pokes required for reward equaled four times the number of rewards earned. The PR4 schedule was ran between 3 and 5 days until the difference in rewards between two consecutive days was three or less. Mice that do not meet this criterion or earned less than five rewards during the PR4 schedule were excluded.

Successfully trained mice were subsequently tested for PR4 and FR1 operant tasks with chemogenetic activation of ^LH^PBN. Vehicle or CNO (1.5 mg/kg in 0.5% DMSO) was intraperitoneally injected in mice in the home cage 30 min before test. Experiments were conducted in the following order on consecutive days: PR4 (saline), PR4 (CNO), FR1 (saline), and FR1 (CNO). Each hourly session was video recorded with an NIR video camera. Nose pokes were recorded by Med-PC software. Reward search, defined as each trip from the nose poke port to liquid dipper port, was automatically scored using the motion tracking option in EthoVision XT 12.

### Overnight fast–refeeding experiments

All fasting-refeeding experiments were performed in PhenoTyper chambers (Noldus) delivering a standard 12-hour light-dark cycle. Mice were initially habituated to phenotypers for 2 days with ad libitum access to water and chow. Chow was removed from chambers at 5:00 p.m. on the fourth day and then returned at 10:00 a.m. the following day to accustom mice to fast-refeed. The 5:00 p.m.–fast 10:00 a.m.–refeed regime was used in all experiments.

For hM3D chemogenetic experiments, vehicle control trials were performed before CNO test trials, and mice were given at least 1 day of rest in between. Vehicle or CNO (1.5 mg/kg in 0.5% DMSO) was intraperitoneally injected 30 min before refeeding, and food intake was subsequently measured for 3 hours. For experiments involving BP, BP (0.1 mg/kg) or saline was intraperitoneally injected in mice an hour before each trial.

For ChR2 optogenetic experiments, opto-off trials were conducted before 20- and 40-Hz opto-on trials, and mice were given at least 1 day of rest in between. Food was returned to chambers 5 min after the start of optostimulation, and a 3-hour food intake was measured. A 460-nm LED light source (Prizmatix) was delivered via 400-μm core, NA 0.48 patch cords connected by rotary joint (Doric Lenses) to fiber optic cannula implanted in mice, and the light power at the fiber tip (200-μm diameter) was calibrated to be 1 to 1.5 mW/mm^2^ at 0.5-mm tissue depth. The LED light source was controlled by a programmable time to live (TTL) pulser (Prizmatix) that receives commands from the EthoVision XT software (Noldus) to deliver light at specific timings and frequencies. Specifically, light was delivered on a 1-s on 3-s off protocol for 3 hours, and 20- and 40-Hz stimulation were delivered in 10-ms pulses during the 1-s on period.

For experiments involving both ChR2 (opto) and hM4D (chemo) components, trials were performed in the following order with at least 1 day of rest in between: opto-off chemo-off (Veh), opto-on chemo-off (Veh), and opto-on chemo-on (CNO). Vehicle or CNO (1.5 mg/kg in 0.5% DMSO) was intraperitoneally injected 1 hour before refeeding, and food intake was measured similarly as above. Feeding bout patterns were measured by PhenoTyper and analyzed by the EthoVision XT 12 software.

For the experiment assessing the effect of ^LH^PBN KORD attenuation on LiCl-induced visceral malaise, mice were housed in a home cage with a custom-built confined food feeder. Mice were overnight fasted before each trial. LiCl [84 mg/kg; 0.20 M at 10 ml/kg; Sigma-Aldrich L9650; adapted from (*31*)] or saline was intraperitoneally injected in mice an hour before food return. At 5 min before food return, mice were subcutaneously injected with SalB (10 mg/kg in DMSO; Sigma-Aldrich) or DMSO. Subsequent 1-hour chow food intake was then measured in each trial.

### Sucrose preference test

The test was performed in PhenoTyper chambers delivering a standard 12-hour light-dark cycle, modified to hold two drinking bottles. Mice were initially habituated for 3 days with ad libitum access to chow and water in both bottles. The sucrose preference test was then conducted as per recommendations to minimize variability (*55*). Mice were first sensitized to 2.5% sucrose in one bottle for 4 days to overcome neophobia. On test days, vehicle or CNO (1.5 mg/kg in 0.5% DMSO) was intraperitoneally injected at the start of the dark cycle (7:00 p.m.; and subsequently every 4 hours for overnight experiments), and sucrose/water consumption was monitored for two time segments, namely, 7:00 p.m. to 10:00 p.m. and then 7:00 p.m. to 9:00 a.m. to account for temporal variations in individual drinking patterns. For all days that mice were provided with sucrose and water (including test days), the positions of the sucrose-containing and water-containing bottles were swapped every day at 9:00 a.m. to avoid confounds due to location preference. Vehicle and CNO test experiments were each performed on two consecutive days with alternating bottle positions. Sucrose and water consumption were monitored by measuring the change in weight of drinking bottles with leakage. Sucrose preference was calculated by taking sucrose intake as a percentage of the total fluid intake.

### Conditioned place preference tests

Chemogenetic conditioned place preference test was performed in a partitioned dual-zone chamber with blue walls in one zone and black and white striped walls in the other zone. A removable barrier divides the two zones to confine mice within specific zones during the conditioning sessions. On the first day, mice were given 15 min of free access across chamber to acclimatize to the environment and also to determine the basal place preference. Over the next 5 days, mice were subjected to daily morning vehicle and afternoon CNO 30-min conditioning sessions, with CNO paired with the more preferred zone. Vehicle or CNO (1.5 mg/kg in 0.5% DMSO) intraperitoneal injections were performed on mice in home cage 30 min before each session, and mice were subsequently confined in the respective zones for 30 min to associate zone-specific wall patterns with paired stimuli. On the final testing day, mice were given 15 min of free access across chamber to determine their conditioned place preference.

Optogenetic conditioned place preference test was conducted in the same chamber. On the first day, optic fiber–implanted mice were fitted with patch cords and given 30 min of free access across chamber to acclimatize to the environment and also to determine the basal place preference. Mice were subjected to daily 30-min sessions of closed-loop optogenetic conditioning over the next 5 days. Mice fitted with patch cords were given free access across two zones, and optostimulation was activated whenever mice entered their basal preferred zone. The optostimulation protocol consists of continuous 40-Hz stimulation delivered in 10-ms pulses. A 460-nm LED optostimulation setup is as described above. On the final test day, mice fitted with patch cords were given 30 min of free access to determine their conditioned place preference.

In both experiments, sessions were video recorded with an overhead monochromatic video camera (Basler), and motion was tracked and analyzed by the EthoVision XT 12 software. Place preference was determined by the percentage time occupancy in each zone.

### Fiber photometry

Photometry recording setup is as follows: 473-nm laser (Omicron) was delivered via 400-μm, core NA 0.48 patch cords connected via rotary joint (Doric) to fiber optic cannula implanted in mice, and the light power at the fiber tip (200-μm diameter) was calibrated to be 1 to 2 μW/mm^2^ at 0.5-mm tissue depth. Light stimulation frequency was determined by an optical chopper (Thorlabs). GCaMPs or GFP fluorescence emission was received by a femtowatt photoreceiver (NewPort), and signals were phase-lock amplified (Stanford Research Systems) and digitized (LabJack), then recorded by a customized Python program based on Fiberkontrol (https://github.com/logang/Fiberkontrol) (*56*).

Acute electric shocks experiments were conducted in sound-attenuated fear-conditioning chambers (Med Associates Inc.). Fiber optic–implanted mouse was attached to patch cords and habituated in the chamber for 1 hour. Random interspersed electric shocks of specific intensity and duration were delivered to the metal grid flooring under Video Freeze software command (Med Associates Inc.), and video recording was done using a high-speed firewire monochrome NIR video camera with NIR in-house lighting. TTL signal outputs were sent from Video Freeze to fiber photometry recording program to indicate timings of electric shocks.

Shock-tone conditioning was conducted in a fear-conditioning chamber with black acrylic A-roof and steel grid flooring, and surfaces were cleaned with detergent with mild lemon scent. Mice were habituated in this chamber for 1 hour 1 day before conditioning and were further habituated for 30 min on conditioning day. Five 20-s pure tones (90 dB, 75 kHz) (CS) that ended with 1-s electric shocks (0.7 mA; US) were delivered to the fear-conditioning chamber over a duration of 10 min. Mice were then given 10-min rest before return to the home cage. Tone extinction was performed the following day in a separate fear-conditioning chamber consisting of white acrylic curved walls and flooring, and surfaces were cleaned with 2% acetic acid solution. Each mouse was attached to patch cords and habituated in the tone extinction chamber for 30 min. Thirty tone extinction trials consisting of 10-s pure tones (90 dB, 75 kHz) were then delivered to the tone extinction chamber. Photometric recordings were conducted at tone extinction trials 1, 2, 9, 10, 19, 20, 29, and 30, and TTL signal outputs were sent from Video Freeze to fiber photometry recording program to indicate tone timings. Mouse freezing behavior was recorded by the NIR video camera and analyzed by Video Freeze.

Acute pinches were conducted on mice lightly anesthetized with 1.25% isoflurane and placed on a platform housed in a fear-conditioning chamber. The experimenter was cued to pinch mice upon hearing a 5-s sound cue commanded by Video Freeze, and pinches were performed by lightly squeezing tail or paws with a blunt tip forceps for 5 s or until a reflex behavior response was triggered.

Tail dips were conducted on mice lightly anesthetized with 1.25% isoflurane and placed on a platform housed in a fear-conditioning chamber. The experimenter was cued to lower the mouse tail into a temperature-controlled water bath for 20 s upon hearing a sound cue commanded by Video Freeze. TTL signal outputs were sent from Video Freeze to fiber photometry recording program to indicate timings of sound cues.

### Graded shocks motion index experiment

The experiment was conducted in a standard fear-conditioning chamber (Med Associates Inc.). A day before the experiment, mice were habituated for 30 min in the chamber. On the day of the experiment, mice were given subcutaneous injections of SalB (10 mg/kg in DMSO) and returned to the home cage. Thirty minutes later, mice were placed in the chamber and subjected to 5-s pulses of graded electric shocks (0, 0.06, 0.12, 0.3, and 0.7 mA) delivered by metal grid flooring with 2-min intervals. Motion tracking was done by Video Freeze (Med Associates Inc.).

### Von Frey paw tactile sensitivity test

Mice were first given two daily sessions on the von Frey platform. The platform consisted of an elevated metal grid with 5-mm perforations and 1-mm border, and each mouse was confined in a transparent observation arena measuring 15 × 10 × 10 cm. On the third and fourth days, mice were given subcutaneous vehicle injections and placed in the observation arena for 30 min before testing with a series of Semmes-Weinstein monofilaments (Aesthesio, Ugo Basile) to determine basal paw tactile sensitivity. On the fifth day, mice were given subcutaneous injections of SalB (10 mg/kg in DMSO) instead to determine the effect of KORD activation in paw tactile sensitivity. Results from the left and right hind paws were averaged. For experiments assessing the effect of hM3D activation, CNO (1.5 mg/kg in 0.5% DMSO) or corresponding vehicle was intraperitoneally injected 30 min before each trial.

### EPM test

The test was performed on a standard EPM (Noldus). Mice were first given intraperitoneal injections of CNO (1.5 mg/kg in 0.5% DMSO) and returned to the home cage for 30 min. Afterward, mice were placed at the center of the platform facing a specific open arm, and their behaviors were recorded for 10 min using a Google Pixel 3 camera at 720p. Motion tracking into the open and closed arms was performed using EthoVision XT 12 software, and automatically generated number of arm transitions were further verified with manual scoring by eye.

### Sustained pinch test

The test was performed according to an earlier published report (*27*). A day before the experiment, mice were habituated in a clear-bottom chamber with an alligator clip (Generic Micro Steel Toothless Alligator Test Clips with Smooth Jawed and Microscopic Tip 5amp; Amazon) for 15 min. On the experiment day, mice were given subcutaneous injections of SalB (10 mg/kg in DMSO) and returned to the home cage for 30 min. Afterward, an alligator clip was applied to the ventral skin between the foot pad and heel of the left hind paw of each mouse, and the mouse was placed in the clear-bottom chamber and a 1-min video was recorded from the bottom using a Google Pixel 3 camera at 720p. Time spent on paw licking was manually scored by eye.

### Nesting behavior experiment

Before the experiment, mice were first habituated for 10 min in a transparent glass cylinder, with the open end shielded by a net and the opposite end occupied by balled-up paper towel commonly perceived as bedding material by mice. Afterward, mice were intraperitoneally injected with CNO (1.5 mg/kg in 0.5% DMSO) and returned to the home cage. Thirty minutes later, mice were placed back in the habituated cylinder, and their behavior was recorded using a Google Pixel 3 camera at 720p for 15 min. Time spent nesting, defined as the action of pulling and fraying of paper towel, was manually scored by eye. Time spent grooming and the breakdown of grooming activities were also manually scored by eye. The percentage of time engaged in active movement was automatically scored by the activity analysis option in EthoVision XT 12, which scores image pixel changes within the experiment arena over time.

### Effect of acute foot shocks on PBN c-fos expression

In this experiment, mice were bilaterally injected with 0.25% Alexa Fluor 555–conjugated CTB in LH for retrograde labeling of ^LH^PBN 1 week before the experiment. Each mouse was individually housed a few days before the experiment to ensure that they do not sustain fight injuries and remain calm. The experiment was conducted in a standard fear-conditioning chamber (Med Associates Inc.). They were then given two daily sessions of 15-min habituation in the chamber. On the day of experiment, each mouse was placed in the chamber and subjected to two 5-s pulses of specified electric shocks (0, 0.06, 0.12, 0.3, and 0.7 mA) delivered by the metal grid flooring. The mouse was then returned to the home cage and perfused 1 hour later for c-fos immunostaining of PBN. For experiments involving BP, BP (0.1 mg/kg) or saline was intraperitoneally injected in mice an hour before each trial.

### Effect of lithium chloride, formalin, and tactile itch on PBN c-fos expression

Mice were bilaterally injected with 0.25% Alexa Fluor 555–conjugated CTB in LH for retrograde labeling of ^LH^PBN 1 week before the experiment. To induce visceral malaise, mice were intraperitoneally injected with either LiCl [84 mg/kg; 0.20 M at 10 ml/kg; Sigma-Aldrich L9650; adapted from (*31*)] or saline and returned to the home cage. They were then perfused 1.5 hour later for c-fos immunostaining of PBN. To induce tactile itch, the nape area was shaved a week earlier, and an adhesive sticker was applied on the nape. Itch induction with adhesive sticker was confirmed by mice scratching the affected area, whereas nonadhesive paper used on control mice did not induce scratching. They were perfused 1.5 hour later for c-fos immunostaining. To induce inflammation, mice were restrained by scruffing, and their left hind paws were dorsally injected with 20 μl of saline or 2% formalin (diluted in saline). Animals were perfused 3 hours after injection for c-fos immunostaining in PBN.

### RNA fluorescence in situ hybridization

Samples were fresh frozen, and the RNAScope Fluorescent Multiplex Assay kit (ACDBio) was used. Mice were deeply anesthetized with ketamine/xylazine mixture and cervical dislocation was performed. Brains were collected, immediately frozen in optimal cutting temperature (OCT) on dry ice, and stored at −80°C. Brains were sectioned at 20 μm onto microscope slides, postfixed in cold 4% paraformaldehyde (PFA) at 4°C for 1 hour followed by a quick wash in phosphate-buffered saline (PBS), and then serially dehydrated in 50, 70, and 100% ethanol. Slides were dried at 40°C for 20 min and permeabilized at room temperature with protease IV provided with the kit. Probe hybridization was done at 40°C for 2 hours. After hybridization, slides were washed in wash buffer provided in the kit, and then incubated in the order of amplification reagents (amp 1 to 4). After incubating with 4′,6-diamidino-2-phenylindole for 3 min, slides were mounted and coverslipped.

RNAScope probes used in the study were *Calb1* (428431), *Calb2* (313641), *CCK* (402271), *CGRP* (420361), *mCherry* (431201), *Pdyn* (318771), *Vglut2* (319171), and *Fos* (316921).

### Immunohistochemistry

Mice were deeply anesthetized with ketamine/xylazine mixture and transcardially perfused with PBS followed by 2 to 4% PFA in 1× PBS. Collected brains were postfixed in 2 to 4% PFA overnight at room temperature. Brains were then washed with PBS and transferred into 15 (4 hours) and 30% sucrose (overnight), respectively, for dehydration. Brains frozen in OCT compound (Tissue-Tek, Sakura) were sectioned at 40 μm in the coronal plane using Leica Cryostat (CM1950). For PBN c-fos quantification, sections were continually collected from AP coordinates, −4.9 to −5.4. Free-floating sections were washed with PBS three times for 5 min and incubated in blocking buffer (5% serum, 3% bovine serum albumin, and 0.4% Triton X-100 in PBS) for 2 hours at room temperature. Sections were then incubated in the respective primary antibodies diluted in blocking solution at 4°C for 24 to 36 hours. After washing in 0.1% Triton X-100 in PBS three times for 5 min, sections were subjected to secondary antibody incubation in blocking solution for 2 hours at room temperature. Sections were finally washed again before being mounted onto microscope slides (Superfrost, Fisherbrand) with Fluoromount-G (Southern Biotech) and coverslipped.

Primary antibodies used were rabbit anti–c-fos (1:500; Cell Signaling Technology, cat. no. 2250, RRID:AB_2247211), goat anti-CGRP (1:500; Abcam, cat. no. ab36001, RRID:AB_725807), and rat–anti-mCherry 16D7 (1:1000; Thermo Fisher Scientific, cat. no. M11217, RRID:AB_2536611).

Secondary antibodies used were Alexa Fluor–coupled donkey anti-rabbit 488 (1:500; Thermo Fisher Scientific, cat. no. A-21206, RRID:AB_2535792), donkey anti-rabbit 647 (1:500; Thermo Fisher Scientific, cat. no. A-31573, RRID:AB_2536183), donkey anti-goat 568 (1:500; Thermo Fisher Scientific, cat. no. A-11057, RRID:AB_2534104), and donkey anti-rat 594 (1:500; Thermo Fisher Scientific, cat. no. A-21209, RRID:AB_2535795).

### Whole-cell patch-clamp recording of acute brain slices

The experiment was performed on acute hypothalamic slices from VGluT2-Cre mice expressing ChR2-mCherry and GFP in glutamatergic PBN and LH neurons, respectively. Mouse brains were rapidly removed after decapitation and placed in high-sucrose ice-cold oxygenated artificial cerebrospinal fluid (ACSF) containing the following: 230 mM sucrose, 2.5 mM KCl, 10 mM MgSO_4_, 0.5 mM CaCl_2_, 26 mM NaHCO_3_, 11 mM glucose, 1 mM kynurenic acid, pH 7.3, 95% O_2_/5% CO_2_. Coronal brain slices were cut at a thickness of 250 μm using a vibratome (VT1200S; Leica Biosystems) and immediately transferred to an incubation chamber filled with ACSF containing the following: 119 mM NaCl, 2.5 mM KCl, 1.3 mM MgCl_2_, 2.5 mM CaCl_2_, 1.2 mM NaH_2_PO_4_, 26 mM NaHCO_3_, and 11 mM glucose (pH 7.3), equilibrated with 95% O_2_ and 5% CO_2_. Slices were allowed to recover at 32°C for half hour and then maintained at room temperature. Whole-cell patch-clamp recordings were performed on GFP-positive glutamatergic neurons in LH. The cells were visualized using a charge-coupled device camera and monitor. Pipettes used for recording were pulled from thin-walled borosilicate glass capillary tubes (length, 75 mm; outer diameter, 1.5 mm; and inner diameter, 1.1 mm; World Precision Instruments) using a DMZ Zietz-Puller (Zeitz). Patch pipettes (2 to 4 megohms) were filled with an internal solution containing 135 mM cesium methanesulfonate, 4 mM MgCl_2_, 10 mM Hepes, 1 mM EGTA (pH 7.2), 0.4 mM Mg–adenosine 5′-triphosphate (ATP), 4 mM Na–guanosine 5′-triphosphate (GTP), and 10 Na_2_-phosphocreatine (pH 7.3 with CsOH; 295 mosmol) for voltage-clamp recording; and an internal solution containing 105 mM K-gluconate, 30 mM KCl, 4 mM MgCl_2_, 10 mM Hepes, 0.3 mM EGTA, 4 mM Na-ATP, 0.3 mM Na-GTP, and 10 mM Na_2_-phosphocreatine (pH 7.3 with CsOH; 295 mosmol) for current-clamp recording. The access resistance, membrane resistance, and membrane capacitance were monitored during the experiment to ensure the stability and the health of cells. To evoke synaptic transmission in ChR2-expressing PBN terminals in LH, 5-ms photostimulation (460 nm) was delivered by LED illumination system (pE-4000), and synaptic responses of the LH neurons were recorded with addition of specific channel blockers into bath of recording chamber.

### Confocal fluorescence microscopy and image processing

All images were acquired in the SBIC-Nikon Imaging Centre at Biopolis (Singapore) with an A1R+ SI confocal microscope (Nikon). Images were processed and analyzed using ImageJ.

### Statistics

Statistical analyses were performed using GraphPad Prism 8 software or Microsoft Excel, and all results are listed in table S1. All results are expressed as means ± SEM, and *P* < 0.05 is considered as significant. All data were first tested for normality using the Shapiro-Wilk test, and then appropriate statistical tests were determined accordingly. All tests were two tailed unless specified. For paired *t* tests, the Wilcoxon matched-pairs signed rank test was used whenever datasets had nonnormal distributions. For unpaired *t* tests, the Mann-Whitney test was used whenever datasets had nonnormal distributions. Unpaired tests with normal distribution but unequal SD were further subjected with Welch’s correction. One-way repeated-measures analysis of variance (ANOVA) was used with the post hoc Holm-Sidak’s multiple comparisons test to determine significant differences between specific trials. Two-way repeated-measures ANOVA was used with the post hoc Sidak’s multiple comparisons test to determine differences between the two mouse groups.

## SUPPLEMENTARY MATERIALS

Supplementary material for this article is available at http://advances.sciencemag.org/cgi/content/full/7/19/eabe4323/DC1

## Appendix

View/request a protocol for this paper from *Bio-protocol*.
